# Midwives’ capacity for improvement of preterm newborns’ health

**DOI:** 10.18332/ejm/147445

**Published:** 2022-05-23

**Authors:** Sevil Hakimi

**Affiliations:** 1 School of Nursing and Midwifery, Research Center of Psychiatry and Behavioral Sciences, Tabriz University of Medical Sciences, Tabriz, Iran

**Keywords:** preterm newborns, midwives

**Dear Editor,**

Preterm birth is a worldwide health challenge. One in ten neonates is born before 37 weeks of gestation. It is a tremendous burden on the health system as well as on families. Approximately 1 million neonates die, following preterm birth complications. Moreover, many survivors face health problems including hearing, visual, cognitive as well as learning impairment^1^. In almost all countries that have valid and reliable data gathering systems, the incidence of preterm birth has been shown to increase over time. Multiple pregnancies, the rising age of pregnancy and the use of new methods of artificial reproductive technology are among the causes of the increasing incidence of preterm births^2^.

Midwives are one of the core elements of the health workforce. They have demonstrated a role in the health of women and newborns. Sandall et al.^3^ in their systematic review showed that midwifery-led care can improve several maternal and newborn outcomes. According to the World Health Organization (WHO), women who received midwifery care by skilled and professional midwifery team members, are 16% less likely to lose their newborns and 24% less likely to experience preterm labor^4^. Although well-designed studies show the positive effect of midwifery-led care on maternal and newborn outcomes, the direct and indirect roles of midwives on preterm newborns’ health has been neglected.

It is suggested that the impairment of the mother’s mental health during the early and late postpartum period and her inability to adjust to the mother’s role, affect mother–infant attachment and the infant’s future abilities^5^. Midwives have a considerable role in supporting mothers during the postpartum period. Despite the introduction and implementation of family-centered care, neonatal intensive care units (NICUs) remain stressful environments for the mothers. The mother after preterm childbirth is vulnerable and mentally fragile. Preterm birth is a traumatic event and considered an important risk factor for postpartum depression. The evidence shows that mothers with preterm childbirth are at higher risk of postpartum depression and even post-traumatic stress disorders than mothers who gave birth at term^6^. Midwives have a unique role in supporting mothers. They can identify parents in the NICUs who have mental health risk factors and provide supportive interventions and implement follow-up after the mother’s discharge. With timely screening and referring as well as encouraging mothers for breastfeeding and via kangaroo care, midwives can play a key role in improving mothers’ mental health. In order to alleviate mothers’ stress, competent midwives can provide counselling with use of different methods including cognitive behavior therapy, mindfulness etc.

The beneficial effect of breastfeeding is well documented. Breast milk may improve brain connectivity and protect the newborn from neurocognitive impairment^7^. Mothers who had preterm childbearing, experienced different emotions including feeling guilty, fear, anger and avoidance. An inverse correlation exists between postpartum depression and breastfeeding. Long-term exclusive breastfeeding may be associated with decreased rates of post-partum depression in adult and adolescent mothers^8^. Re-hospitalization of preterm newborns owing to feeding problems is a common challenge with a substantial burden on the health system and families^9^. Promoting breastfeeding is an integral part of a midwife’s role.

Kangaroo mother care (KMC) is a healthcare strategy for premature and low birth weight infants that includes skin-to-skin contact between mother and infant. It is a highly effective, low-cost intervention that forms part of the care for small newborns across the globe. In low- and middle-income countries, effective affordable care is necessary for reducing neonatal morbidity and mortality, especially with regard to premature infants^10^. WHO recommends the implementation of KMC for all stable preterm newborns. Recently, the WHO immediate KMC working group published an article which showed that immediate Kangaroo care is an effective method for reducing neonate mortality^11^.

Nearly 80% of preterm births occur in the less developed countries^2^. Apart from skilled workforce and budget shortages, these countries face inefficient data registry systems. Midwives can be considered a valuable resource for assistance with data organization and recording, in every setting. Midwives can provide safe, stable, nurturing relationships between mother and newborn and alleviate parents’ emotional disorders. By providing exclusive breastfeeding and kangaroo care, midwives can support optimum growth and development of preterm newborns. Midwifery education needs re-orientation to include courses on preterm newborns in undergraduate as well as postgraduate midwifery programs.

Investing in midwifery education and enhancing the abilities of the midwifery workforce, can improve the next generation’s health.

## Data Availability

Data sharing is not applicable to this article as no new data were created.
